# Efficacy and safety of intraurethral Erbium:YAG laser treatment in women with stress urinary incontinence following failed intravaginal laser therapy: a retrospective study

**DOI:** 10.1007/s10103-023-03872-5

**Published:** 2023-09-09

**Authors:** Yung-Ling Tseng, Chi-Feng Su

**Affiliations:** 1 Department of Education, Kuang Tien General Hospital, Taichung, Taiwan; 2 Department of Gynecology and Obstetrics, Kuang Tien General Hospital, No.117, Shatian Road, Shalu District, Taichung, 433 Taiwan

**Keywords:** Erbium:YAG laser, Stress urinary incontinence, Genitourinary syndrome of menopause, Intraurethral laser

## Abstract

Urinary incontinence (UI) is a prevalent condition affecting 25–45% of women and is linked to factors such as menopause, parity, high body mass index, and radical pelvic surgery. Among the three types of UI, stress incontinence (SUI) is the most common, accounting for almost 50% of cases, followed by urgency and overflow incontinence. UI has been found to be associated with reduced quality of life and mental stress. Non-invasive laser treatment is the safest and most effective option for managing SUI, with intraurethral Erbium SMOOTH^TM^ laser treatment holding promise for patients experiencing SUI even after undergoing previous failed intravaginal Erbium:YAG laser treatment. The study recruited 93 female patients with mild to moderate SUI who had received two courses of intravaginal Erbium:YAG laser between January 2015 and June 2018. Of these, 22 patients (23%) who continued to experience SUI after a four-week interval for a second intravaginal Erbium:YAG laser were selected for intraurethral laser treatment in January 2019. The efficacy of the treatment was evaluated by comparing the pre- and post-treatment ICIQ-UI SF score. The urethral length was measured before the procedure. The main procedure involved delivering non-ablative laser energy using Erbium SMOOTH^TM^ technology 2940 nm via a 4-mm cannula with personalized length and fluence was 1.5 J/cm. The 22 female patients with persistent SUI received intraurethral Erbium:YAG laser treatment. Their average age was 47.5 years, with an average of 2 parities and a mean body mass index of 20.97. All patients completed the ICIQ-SF questionnaire before and 3 months after the procedure. Of the patients, 77% reported improvement in symptoms, with 6 reporting strong improvement and 11 reporting improvement. The treatment was well-tolerated, with mild and transient adverse effects such as urinary infection in 1 patient (4.5%) and mild pain in 7 patients (31.8%). Intraurethral laser treatment may be helpful for Taiwanese women with persistent SUI after vaginal laser treatment. However, patients with prior pelvic surgery or pelvic organ prolapse history may limit the efficacy of intraurethral laser. Additional research is necessary to comprehensively investigate the advantages of intraurethral laser therapy. However, using intraurethral Erbium SMOOTH^TM^ laser treatments to rejuvenate tissues and enhance structural support could be a promising avenue for managing stress urinary incontinence in Taiwanese women.

## Introduction

Urinary incontinence (UI) affects 25% to 45% percentage of women, with stress urinary incontinence (SUI) being the most prevalent type [1]. SUI is defined as the unintentional discharge of urine during activities that increase intraabdominal pressure, such as coughing, laughing, or sneezing, due to weakened muscles around the urethra that are unable to maintain a secure seal. Aging, smoking, obesity, radical pelvic surgery, menopause, pregnancy, and method of delivery are some of the known risk factors for SUI [2].

Genitourinary syndromes of menopause (GSM)-associated estrogen deficiency is linked to urinary incontinence, as it leads to alterations in the genitourinary tract and heightens the likelihood of developing the condition. The prevalence of stress urinary incontinence (SUI) increases with age, affecting up to 50% of women [3]. The condition can have a significant impact on mental health, well-being, and quality of life, often resulting in physical discomfort, embarrassment, social isolation, and avoidance of certain activities or situations, including sexual function [4]. The association between SUI, falls, and pathological fractures has also been noted [5]. Therefore, addressing SUI remains an important issue to alleviate mental stress and improve the overall quality of life of women.

Lifestyle and behavioral change with Pelvic floor muscle training (PFMT) are universally recommended as first-line nonpharmacological management of SUI [6, 7]. Pelvic floor muscle training involves the strengthening of the muscles that support the bladder and urethra and can be done through a variety of exercises such as Kegels. However, mild improvement on quality of life (QoL) scale of SUI patient performing PFMT was reported, with no significant change in pad test when comparing PFMT to no treatment [8]. Supervised Kegel exercise with biofeedback has been found to be effective in improving the incontinence severity index (ISI) score. However, some studies have not found significant improvement in pelvic floor muscle strength with this intervention [9]. The overall effectiveness of PFMT to reduce urinary incontinence has been found to vary widely, with studies reporting effectiveness rates ranging from 27% to 75%, depending on the duration and intensity of the exercise program, and how correctly the exercises are performed [9]. Several non-estrogenic way has been suggested to manage the urogynecological symptoms caused by GSM, to avoid the potential adverse effect of hormone therapy [10]. Surgical management with a sub-urethral sling or other suspension methods has shown promising success rates but may have long-term complications [11].

Intravaginal Erbium:YAG laser treatment has shown promising results as a safe and effective treatment for improving symptoms of stress SUI in several studies [12, 13]. In patients who have previously undergone invasive procedures for SUI and have not experienced satisfactory results, intravaginal laser treatment has shown to improve their clinical symptoms [13]. However, the effectiveness of intravaginal laser treatment for stress urinary incontinence (SUI) may vary depending on the type of SUI. Some studies have reported that this treatment is more effective in improving symptoms of SUI types I and II than in SUI type III [14].

Our study aims to address the gap in the literature regarding the potential benefits and limitations of intraurethral laser treatment for stress urinary incontinence. While there are few publications on this topic, our research aims to provide a comprehensive analysis of the available data to determine the effectiveness and safety of this novel treatment option in Taiwanese population.

## Material and method

This is a retrospective study that included 93 female patients with mild to moderate stress urinary incontinence (SUI) who had completed two courses of intravaginal Erbium:YAG laser treatment between January 2015 and June 2018. Of these patients, 22 (23%) who continued to experience persistent SUI after a 4-week interval following the second intravaginal Erbium:YAG laser treatment were selected to receive intraurethral laser treatment in January 2019. The effectiveness of the intraurethral Erbium SMOOTH^TM^ laser treatment was evaluated by comparing the pre- and post-treatment ICIQ-UI SF score. This score measures the degree of distress caused by daily activities affected by UI, and is classified as *no change, improvement* (a decrease of ICIQ-SF score of 1 to 5), or *strong improvement* (a decrease of ICIQ-SF score greater than 5).

**Fig. 1:**
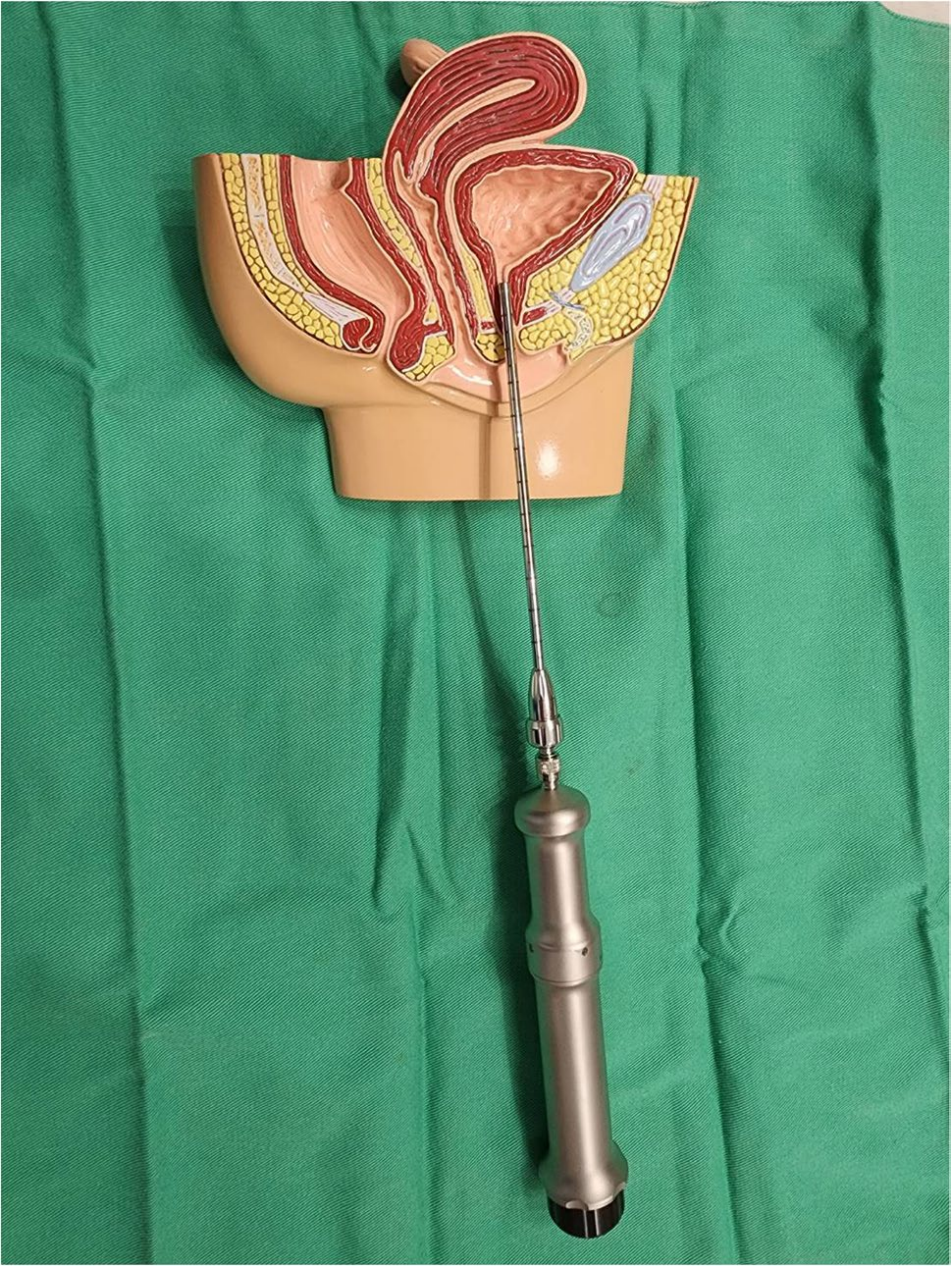
Presentation on the use of the Erbium:YAG laser handpiece for the treatment of SUI

The procedure for intraurethral Erbium SMOOTH^TM^ laser treatment described involved measuring the urethral length by indwelling a Foley catheter and drawing a line at the urethral opening, followed by delivering non-ablative laser energy using Erbium SMOOTH^TM^ technology 2940 nm through a 4-mm cannula with a personalized length as shown in Fig. 1. The pulses were set at a frequency of 1.4 Hz, and the energy was delivered stepwise from the proximal urethra to the urethral orifice at an interval of 2.5 mm. The fluence was 1.5 J/cm^2^. The patient received the procedure at an outpatient clinic without anesthesia. No pre-laser medication was applied, and post-laser medication consisting of cephalexin and phenazopyridine was given for three days to prevent infection.

## Result

### ICIQ-UI SF

The study included 93 female patients with mild to moderate SUI who had completed two courses of intravaginal Erbium:YAG laser from January 2015 to June 2018, with an average age of 47.5 years (range 32–62), an average of 2 parities (range 0–4), a mean body mass index of 20.97(range18.14-28.51), and a mean ICIQ-SF score 10.95(range 4–18) (Table 1).

**Table 1:** Demographic data of 22 patients selected from two previous failed vaginal laser treatments for urinary incontinence

	Average
Age	47.5 (range 32–62)
Parities	2 (range 0–4)
Body mass index (BMI) ICIQ-SF	20.97 (range 18.14–28.51) 10.95 (range 4–18)

**Fig. 2:**
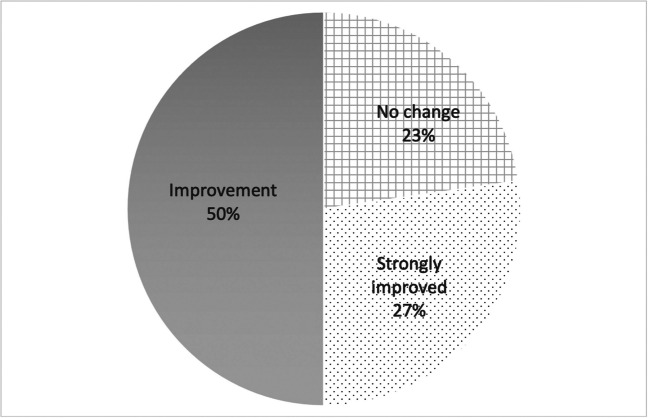
Pie chart of improvement ratio after intraurethral Erbium:YAG laser treatment

All patients completed the ICIQ-SF questionnaire before and 3 months after the procedure, regardless of their urodynamic study classification. Of the 22 patients selected for intraurethral laser treatment, 17 (77%) reported an improvement in symptoms by ICIQ-SF score, including 6 patients reporting strong improvement and 11 patients reporting improvement (Fig. 2).

Five patients did not report any improvement, and the possible reasons for treatment ineffectiveness are reported in Table 2.

**Table 2:** Related medical and operation history of patients without improvement after intraurethral laser therapy

	Pre-ICIQ	Post ICIQ	Change from baseline	Related medical and operation history
P1	9	9	0	Anterior and posterior repair
P2	14	14	0	Anterior and posterior repair
P3	16	16	0	Pelvic surgery
P4	18	18	0	No operation history Cystocele stage 2
P5	9	9	0	Cesarean section

### Adverse effect

**Fig. 3:**
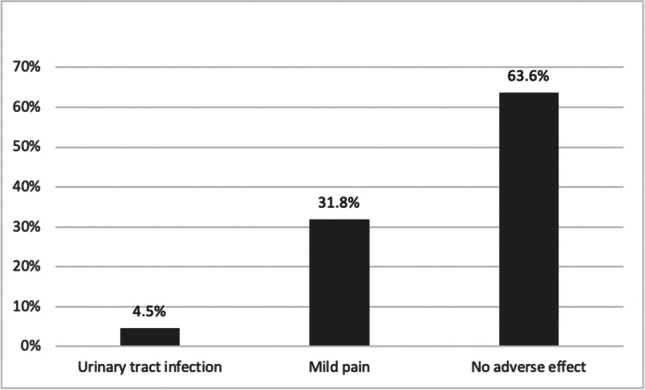
Bar chart of adverse effect after intraurethral Erbium:YAG laser treatment

All patients tolerated the therapy well. Adverse effects following treatment were mild and transient and mostly resolved in three to four days including: 1 patient (4.5%) had urinary infection, which was treated with antibiotics, and 7 patients (31.8%) reported mild pain following the procedure (Fig. 3).

## Discussion

Similar to the promising results of intravaginal Erbium:YAG laser treatment, intraurethral Erbium:YAG laser treatment has also been shown to be a safe and effective method for reducing symptoms of stress urinary incontinence [15]. Non-ablative Erbium:YAG laser showed a non-inferior result while compared to invasive urethral sling surgery [16]. Our results are consistent with a study published by Gasper in 2017, which showed a similar rate of symptom relief in 72% of patients [17]. The improvement was reported stable in both short-term and long-term follow-up [10, 12].

The pathophysiology of UI may play a critical role, particularly in patients who have previously failed intravaginal laser treatment for urinary incontinence. SUI has been linked to bladder neck and urethral incompetence, which can be attributed to several factors, such as levator ani muscle dysfunction and dense connective tissue originating mainly from the vagina [18]. Laser treatment has been reported for vagina rejuvenation with increased collagen, increased in the thickness of the vagina epithelium, and extracellular matrix production [19]. A fractional CO2 laser meliorated vaginal sexual function by increased whole-layered thickness of vagina tissue [20]. Erbium:YAG laser therapy also demonstrated superiority to topical estrogen in vagina mucosa tropism, angiogenesis, and restructuring of lamina propria [21, 22]. The tissue regeneration might be the key to SUI symptom improvement.

The use of intraurethral laser with Erbium SMOOTH^TM^ technology may have a similar rejuvenating effect on the urethra as intravaginal laser treatment has on the vagina. The non-ablative energy from the laser is believed to increase support and structural integrity of the urethra by inducing vasodilation and collagen regeneration. However, more research is needed to provide higher levels of evidence in this field.

A small number of patients in our study did not experience symptom improvement after the procedure. This may be due to periurethral fibrosis-induced intrinsic sphincter deficiency (ISD), as reported in previous studies, which could be a significant factor contributing to the failure of intraurethral laser treatment [17]. ISD may result from damage to innervation following child-delivered injuries, urethral pathologies, prior pelvico-vaginal surgery, or irradiation [23]. However, the efficacy of intraurethral Erbium SMOOTH^TM^ laser treatment seems to be limited in patients with damage-related intrinsic sphincter deficiency (ISD), as compared to those with atrophy-related ISD, as suggested by previous studies [17].

Our study demonstrated that intraurethral Erbium:YAG laser treatment can be effective in patients who have not responded to previous management for SUI. Moreover, in patients who have undergone surgical treatment but still experience SUI, the treatment showed promising results in improving scar tissue and promoting the formation of new collagen fibers [13]. Compared to intravaginal therapy, intraurethral therapy can directly induce regeneration of the urethral tissue, thereby improving the structural integrity of the urethra. However, our study has some limitations. We recruited only a small sample size, which may need further study with a larger sample size to evaluate. The lack of a control group also limits the ability to compare the effectiveness of intraurethral laser treatment with other treatments or no treatment. Therefore, intraurethral laser therapy may be a promising direction in SUI management, especially for patients with failed surgical or nonsurgical treatments, but further research is necessary to confirm its efficacy.

### Limitation

There are several limitations in this study. Firstly, the limited number of cases may affect the generalizability of the findings. Additionally, the study focuses on women who have received a singular intraurethral laser treatment session, which might not fully capture the potential benefits of multiple interventions. In this regard, enhancing treatment frequency could conceivably yield more favorable stress urinary incontinence outcomes—a direction that warrants further research exploration. These limitations should be taken into consideration when interpreting the results of this investigation.

## Conclusion

Our findings suggest that intraurethral laser therapy is an effective treatment option for persistent SUI in Taiwanese women who have undergone previous intravaginal laser procedures, demonstrating a 77% success rate. However, the efficacy of intraurethral laser treatment may be limited in individuals with a history of pelvic organ prolapse or prior pelvic surgery. Further investigations through randomized, large-scale studies are necessary to confirm the potential advantages of using intraurethral Erbium SMOOTH^TM^ laser therapy to rejuvenate tissues and improve structural support for the management of stress urinary incontinence in Taiwanese women. Nonetheless, this treatment approach appears to hold promise for the management of SUI.

## Data Availability

The data that support the findings of this study are available from the corresponding author upon reasonable request.
